# MiR-92a Inhibits the Progress of Osteosarcoma Cells and Increases the Cisplatin Sensitivity by Targeting Notch1

**DOI:** 10.1155/2018/9870693

**Published:** 2018-06-07

**Authors:** Quanxiang Liu, Yang Song, Xianliang Duan, Yuan Chang, Jianping Guo

**Affiliations:** Department of Orthopedics, The Affiliated Hospital of Beihua University, Jilin, China

## Abstract

**Background:**

MicroRNAs (miRs) have been implicated in the development and progression of osteosarcoma. Here, we aimed to illustrate the important role of miR-92a on the regulation of OS development which may help to establish a novel strategy for OS diagnosis and treatment.

**Materials and Methods:**

Cell viability was measured by 3-(4,5-dimethylthiazol-2-yl)-2,5-diphenyltetrazolium bromide (MTT) assay. Cell cycle and apoptosis were assessed by flow cytometry with PI and PI/Annexin-V stain, respectively. The expression of proteins was examined by western blot. qPCR was used to detect the expression of RNA. Cell migration was assayed with transwell assay.

**Results:**

MiR-92a inhibited the proliferation and the migration of OS* in vitro* and reduced the volume of the tumour* in vivo*. Further, miR-92a enhanced cisplatin sensitivity of OS. MiR-92a directly targeted Notch1.

**Conclusion:**

Together, our results indicate that miR-92a inhibited cell growth, migration, and enhanced cisplatin sensitivity of OS cell by targeting Notch1.

## 1. Introduction

Osteosarcoma (OS) is the most common primary malignant tumour in adolescents and children, accounting for approximately 5% of cancer-related deaths worldwide [1, 2]. OS is highly aggressive and accounts for nearly 50% of metastatic bone sarcomas [3]. In recent years, considerable progress has been made in the development of therapeutic strategies of OS, such as combination chemotherapy, radiotherapy, and surgical resection. However, effective strategies for OS remain elusive and the prognosis of advanced OS remains poor [4]. Even after combination chemotherapy, the 5-year survival rate for children diagnosed with OS is less than 53.9%, which can be primarily attributed to chemoresistance [5]. Thus, studies of the molecular mechanism underlying the initiation, development, and drug resistance of OS are urgently needed, as they may provide novel strategies to improve the survival and quality of life of OS patients.

Numerous studies have identified diverse genetic alterations in OS, including tumour suppressor gene mutations and epigenetic modifications [6]. MicroRNAs (miRs) are a class of highly conserved small, noncoding RNAs that attenuate gene expression by binding to the 3′-untranslated regions (3′-UTRs) of target genes and inhibiting mRNA translation or inducing mRNA degradation [7]. MiRs have been implicated in the pathogenesis of various human diseases, including cancer. More than 1000 miRs have been reported to impact the initiation and progression of many kinds of cancer, including OS. In addition, miRs also contribute to the regulation of drug resistance [8–10]. For example, miR-184, miR-138, miR-125, miR-30a, and miR-146b-5p have all been shown to be involved in the development of chemoresistance in OS [11–15].

Deregulation of miR-92a has been implicated in a variety of human malignancies, including gastric, colon, cervical, and breast cancer, resulting in the deregulation of target gene expression [16–19]. Recently, miR-92 was found to be expressed in OS aberrantly [20]. However, little was known about the functions of miR-92a in OS and drug resistance.

In the present study, we first demonstrated the aberrant expression of miR-92a in OS specimens and cell lines. Then, gain- and loss-of-function studies and xenograft models were utilized to explore and validate the function of miR-92a in OS. Moreover, we predicted and confirmed that miR-92a directly targets Notch1.

## 2. Materials and Methods

### 2.1. Patient Tissue Samples

The present study was approved by the Institutional Ethics Committee of Affiliated Hospital of Beihua University. Paired tumour/nontumour OS tissue samples were obtained from 25 patients. The samples were snap-frozen in liquid nitrogen and stored at -80°C after RNA extracting. Informed consent was obtained from each patient to approve the use of their tissues for research purposes.

### 2.2. Cell Lines

MG63, HOS, and hFOB cell lines were purchased from the Cell Bank of the Chinese Academy of Sciences (Shanghai, China). All cell lines were cultured in RPMI-1640 (Hyclone, UT, USA) with 10% FBS (Gibco, CA, USA) and 100 U/mL penicillin (Sigma-Aldrich, St. Louis, MO, USA) under standard cell culture conditions (37°C, 5% CO_2_).

### 2.3. RNA Isolation and Quantitative Real-Time PCR

Total RNA was extracted from cultured cell lines using an RNAiso kit (Takara, China) according to the manufacturer's instructions and reverse transcribed using a PrimeScript RT Reagent Kit (Takara, China). The cDNAs were subjected to qRT-PCR using SYBR Premix Ex Taq (Takara, China) to detect miR-92a and Notch1 mRNA. The relative expression levels of miR-92a were normalized to those of U6 small nuclear RNA (U6-snRNA). GAPDH mRNA was calculated with the 2^-ΔΔCt^ method.

### 2.4. Cell Viability and Clonogenic Assays

Cell viability was evaluated in accordance with the methyl thiazolyl tetrazolium (MTT, Amresco, Solon, OH, USA) method. MG63 and HOS cells were seeded in 96-well plates at 2×10^4^ cells/mL and cultured for the indicated times. After treatment, cells were incubated with 10 *μ*L of 0.5% MTT solution for 4 h at 37°C. Then, the supernatant was discarded, and 150 *μ*L of dimethyl sulfoxide was added to each well. The 96-well plates were shaken for 5 min until the crystals dissolved completely. The absorbance measured at a wavelength of 490 nm using a microplate reader (Bio-Rad Laboratories, CA, USA). All of the experiments were repeated three times. For the clonogenic assay, the cells were resuspended in RPMI-1640 medium supplemented with 10% FBS and seeded in 6-well plates at a density of 300 cells per well. After 12 days, the colonies on the plates were fixed with methanol for 15 min and stained with 0.1% crystal violet for 20 min. Stained colonies were imaged and enumerated.

### 2.5. Western Blotting

MG63 and HOS cells were lysed (20 mM Tris, pH 7.5, 150 mM NaCl, 1% Triton X-100, sodium pyrophosphate, *β*-glycerophosphate, EDTA, Na_3_VO_4_, and leupeptin; Beyotime, Nanjing, China) at 4°C for 30 min. A BCA protein assay kit (Beyotime, China) was used to calculate the total protein concentration in each lysate. Equal amounts of protein were resolved on 4–20% gradient sodium dodecyl sulfate (SDS) polyacrylamide gels and transferred to PVDF membranes by voltage gradient transfer. The membranes were blocked with skim milk for 60 min at RT and incubated with anti-Notch1 and anti-GAPDH primary antibodies (Cell Signalling Technology, Danvers, MA, USA) overnight at 4°C. The membranes were then washed in TBS-T three times and incubated with the corresponding HRP-conjugated secondary antibodies for 120 min. Proteins were detected by chemiluminescence using an ECL kit (Beyotime, Nanjing, China).

### 2.6. Flow Cytometric Analysis of Cell Cycle Profiles

Cells were trypsinized, washed with PBS, and fixed with ethanol. After fixation, the cells were washed again and stained with propidium iodide (PI) for 30 min in the dark. MG63 and HOS cell samples were analysed on a FACSVerse flow cytometer, and the data were analysed with FlowJo software.

### 2.7. Cell Migration Assay

The migratory capacities of MG63 and HOS cells were determined using a transwell Boyden Chamber (8-mm pore size, BD Biosciences) assay. Cells were plated in the upper chambers of Matrigel-coated wells in 24-well plates and incubated for 24 h, whereas medium containing 20% FBS was added to the lower chambers as a chemoattractant. Noninvading cells were removed, and cells adhering to the bottoms of the membranes were fixed with methanol, stained with crystal violet, and counted under an inverted microscope (Nikon, Japan).

### 2.8. Luciferase Activity Assay

The 3'UTR sequence of Notch1 containing putative binding sites for miR-92a was cloned into the pGL3 reporter vector. A 3'UTR with a mutated miR-92 binding site was also generated using fusion PCR and cloned into the pGL3 reporter vector. Cells were plated in 96-well plates and transfected with miR-92a or miR-NC along with the wild-type or mutant Notch1-3'UTR vector. Control cells were transfected with pRL-TK. Cells were harvested 48 h after transfection, and luciferase activity was measured using a Dual-Luciferase Assay Kit (Promega, CA, USA).

### 2.9. Immunohistochemistry

Formalin-fixed tumour tissues were embedded and sectioned into 5-*μ*m-thick sections. After deparaffinization and rehydration, sections were incubated in a 3% H_2_O_2_ solution for 20 min to block endogenous peroxidases. Sections were then incubated with rabbit polyclonal antibodies against Notch1 (Abcam, Cambridge, England) at 4°C overnight. After incubation with HRP-conjugated secondary antibodies (Abcam, Cambridge, England) at 37°C for 2 h, sections were washed, counterstained with diaminobenzidine (DAB), and visualized under a microscope (Nikon, Japan).

### 2.10. Mouse Xenograft Model

A total of 2 ×10^5^ MG63 cells stably expressing miR-92a or a control were injected subcutaneously into the dorsal flanks of nude mice. Tumour volumes and body weights were measured every 2 days beginning 10 days after injection. Tumour volumes were calculated using the following formula: tumour volume =1/2×(length×width^2^).

### 2.11. Statistical Analysis

Data are expressed as the mean ± standard deviation (SD). Comparisons of multiple groups were performed using one-way analysis of variance (ANOVA) followed by Tukey's multiple comparison test. Values of P<0.05 were considered statistically significant. Assays were performed at least three times.

## 3. Results

### 3.1. MiR-92a Was Downregulated in OS Tissues

**Figure 1:**
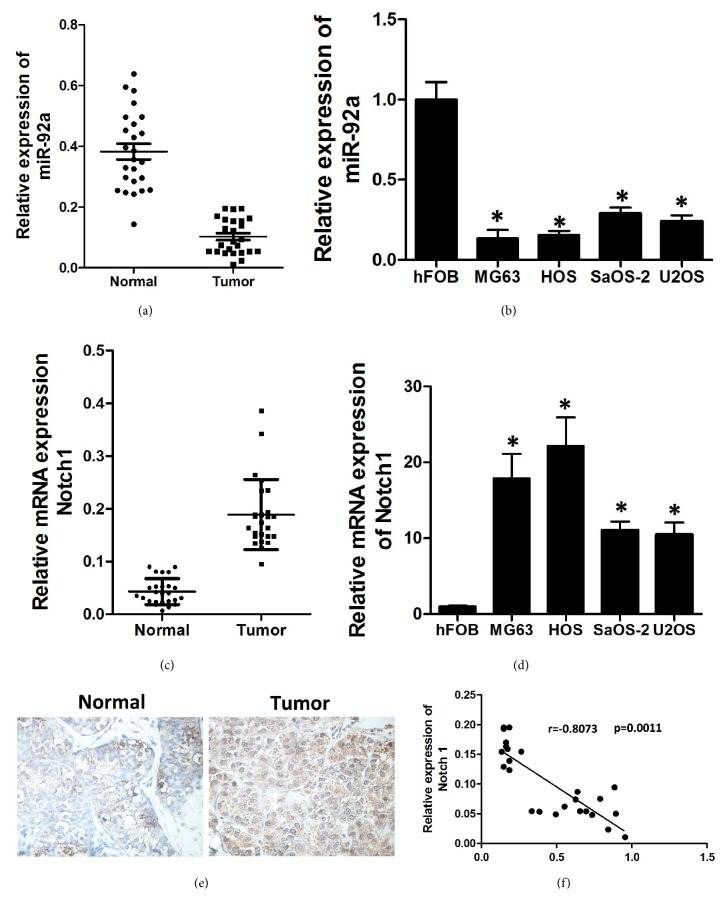
MiR-92a is significantly downregulated in osteosarcoma tissues and cell lines and is negatively correlated with the expression levels of Notch1. (a, b) The expression of miR-92a in osteosarcoma tissues and cell lines. (c, d) The expression of Notch1 in osteosarcoma tissues and cell lines. (e) Immunohistochemistry was used to detect the Notch1 expression in osteosarcoma tissues and the paired adjacent tissues. (f) MiR-92a had a negative correlation with Notch1 according to Pearson Correlation Coefficient. Data represent mean ± SD. *∗*P < 0.05; *∗∗*P < 0.01.

Quantitative real-time PCR (qRT-PCR) was used to detect the expression of miR-92a in clinical tissues and cells. We found that miR-92a was significantly downregulated in OS tissues and cell lines compared to adjacent normal tissues and hFOB cells (Figures 1(a) and 1(b)). Notch1 was significantly upregulated in osteosarcoma tissues and cell lines and was negatively correlated with the expression levels of miR-92a. Meanwhile, qRT-PCR was used to detect the level of Notch1 expression in clinical tissues and cells. We found that Notch1 was significantly upregulated in OS tissues and cell lines compared to adjacent normal tissues and hFOB cells (Figures 1(c)–1(e)). Next, we evaluated the correlation between miR-92a and NOTCH1 and found that the level of miR-92a exhibited a significant negative correlation with the level of NOTCH1 (Figure 1(f)).

### 3.2. MiR-92a Inhibited the Tumourigenesis of OS* In Vitro*

**Figure 2:**
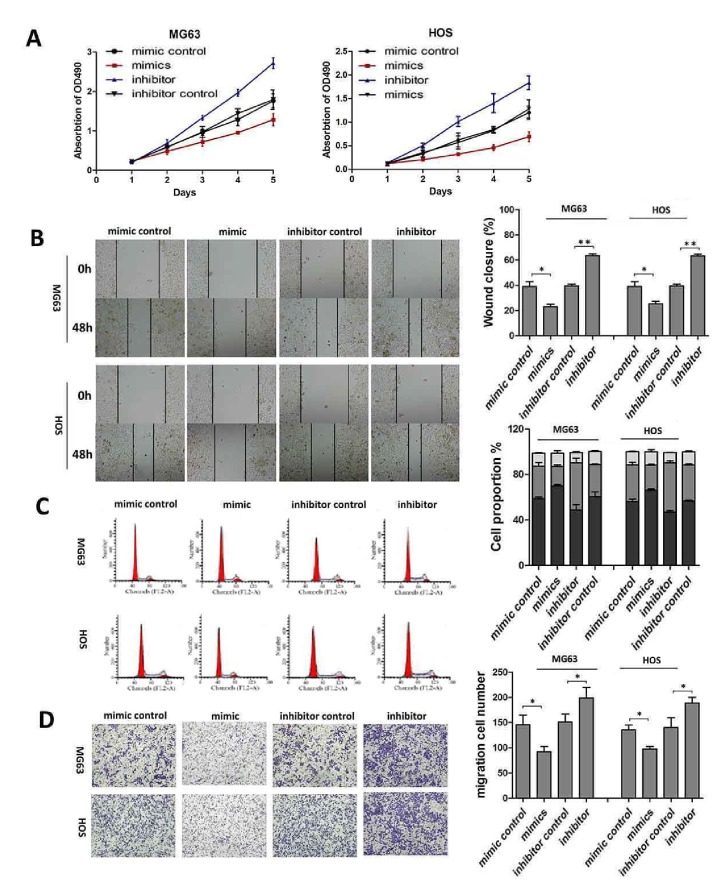
MiR-92a attenuates the tumourigenesis of osteosarcoma in vitro. (A) MTT assay was performed to study the effects of miR-92a on OG63 and HOS cells 1, 2, 3, 4, and 5 days after transfection of miR-92a mimics, mimic control, miR-92a inhibitor, and inhibitor control. (B) Wound healing assay of the OG63 and HOS cells transfected with miR-92a mimics, mimic control, miR-92a inhibitor, and inhibitor control. (C) Flow cytometry was used to detect the cell cycle of OG63 and HOS cells transfected with miR-92a mimics, mimic control, miR-92a inhibitor, and inhibitor control. (D) Transwell assay was used to evaluate the cell migration of of OG63 and HOS cells transfected with miR-92a mimics, mimic control, miR-92a inhibitor, and inhibitor control. Data represent mean ± SD. *∗*P < 0.05; *∗∗*P < 0.01.

To investigate whether reduced miR-92a is important in the development of OS, we performed loss- and gain-of-function assays. We found that overexpression of miR-92a by transfection with miR-92a mimics inhibited the proliferation, migration, and clonogenicity of OS cells and markedly induced cell cycle arrest compared with the negative control. By contrast, depletion of miR-92a significantly promoted the proliferation, migration, and clonogenicity of OS cells, in addition to accelerating cell cycle progression (Figures 2(A)–2(D)).

### 3.3. MiR-92a Overexpression and Notch1 Knock Down Enhanced and Suppressed, Respectively, the Susceptibility of Osteosarcoma Cells to Cisplatin

**Figure 3:**
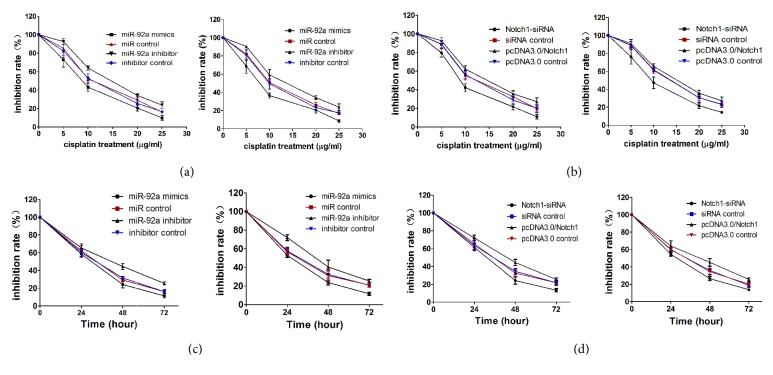
MiR-92a overexpression and Notch1 knock down enhanced and suppressed, respectively, the susceptibility of osteosarcoma cells to cisplatin. (a, b) MTT was used to detect the cell viability of OG63 and HOS cells treated by cisplatin of different concentrations. (c, d) MTT was used to detect the cell viability of OG63 and HOS cells treated by cisplatin of different times. Data represent mean ± SD. *∗*P < 0.05; *∗∗*P < 0.01.

As shown in Figure 3, miR-92a increased the susceptibility of OS cells to cisplatin compared to the miR control. However, a miR-92a inhibitor decreased it (Figures 3(a) and 3(c)). Subsequently, we investigated the effect of Notch1 overexpression or depletion on the responses of OS cells to cisplatin treatment. As expected, overexpression of Notch1 decreased the susceptibility of OS cells to cisplatin, whereas a Notch1 siRNA decreased it (Figures 3(b) and 3(d)).

### 3.4. MiR-92a Directly Targeted NOTCH1

**Figure 4:**
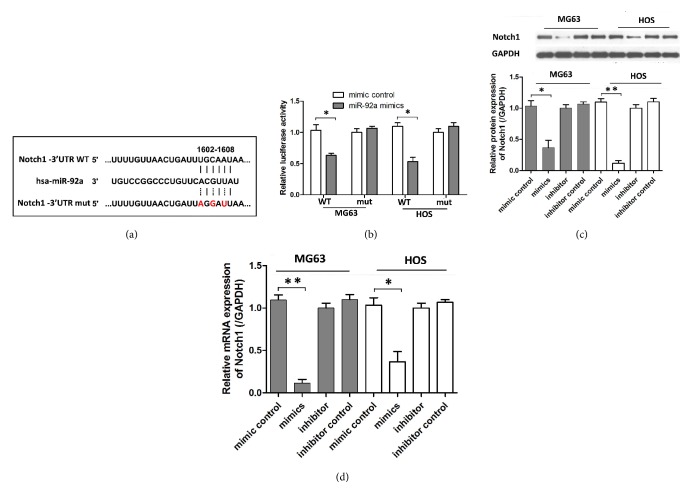
MiR-92a directly targets Notch1 in osteosarcoma cell. (a) A predicted schematic of the seed region of miR-92a in the 3'-UTR of Notch1 and the mutated 3'-UTR of Notch1 by bioinformatics. (b) Relative luciferase activity of the wild-type or mutant pGL3-Notch1-3′-UTR plasmid in OG63 and HOS cells cotransfected with miR-92a mimic or negative control. (c) The protein expression of Notch1 was detected by western blotting. (d) The mRNA expression of Notch1 was detected by qRT-PCR. Data represent mean ± SD. *∗*P < 0.05; *∗∗*P < 0.01.

We performed bioinformatic analyses to identify the target genes of miR-92a. We found that NOTCH1 mRNA contained a putative miR-92a binding site within its 3'UTR region (Figure 4(a)). A luciferase assay was then performed to verify whether miR-92a targets NOTCH1. As shown in Figure 4(b), miR-92a significantly reduced the luciferase activity of the reporter vector carrying the wild-type NOTCH1 3'UTR region. However, this suppressive effect was abolished when the miR-92a binding site in the Notch1 3'UTR was mutated. We further validated these findings by examining Notch1 expression at the protein and mRNA levels. Following overexpression of miR-92a, the endogenous levels of Notch1 protein and mRNA were remarkably downregulated in both MG63 and HOS cells (Figures 3(c) and 3(d)).

### 3.5. Restoration of NOTCH1 Attenuated the Effects of miR-92a in OS

**Figure 5:**
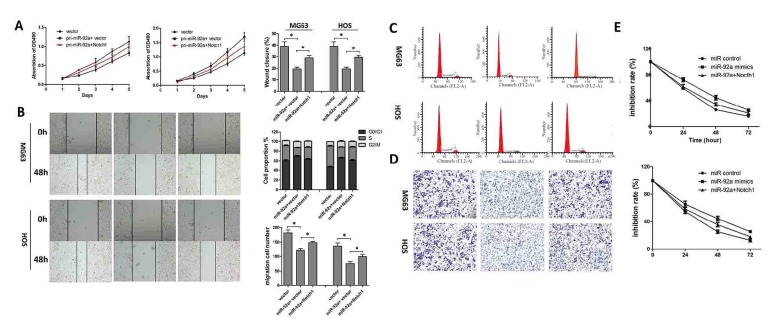
Restoration of NOTCH1 attenuated the effects of miR-92a in osteosarcoma. (A) MTT assay was performed to study the effects of miR-92a on OG63 and HOS cells of different treatments. (B) Wound healing assay of the OG63 and HOS cells of different treatments. (C) Flow cytometry was used to detect the cell cycle of OG63 and HOS cells of different treatments. (D) Transwell assay was used to evaluate the cell migration of OG63 and HOS cells of different treatments. Data represent mean ± SD. *∗*P < 0.05; *∗∗*P < 0.01.

Cells were cotransfected with a NOTCH1 overexpression vector and miR-92a mimic or a control vector and miR control. An MTT assay was used to investigate the viability of OS cells and revealed that the suppressive effects of miR-92a on cell proliferation and migration were significantly reversed by NOTCH1 in MG63 and HOS cells (Figures 5(A), 5(B), and 5(D)). We next analysed cell cycle using flow cytometry. These analyses indicated that Notch1 inhibited the effect of miR-92a on G0/G1 cell cycle arrest (Figure 5(C)). In addition, a chemosensitivity assay indicated that Notch1 attenuated the effects of miR-92a on the sensitivity of OS cells to cisplatin (Figure 5(E)).

### 3.6. MiR-92a Inhibited OS Tumourigenesis and Enhanced Cisplatin Sensitivity* In Vivo*

**Figure 6:**
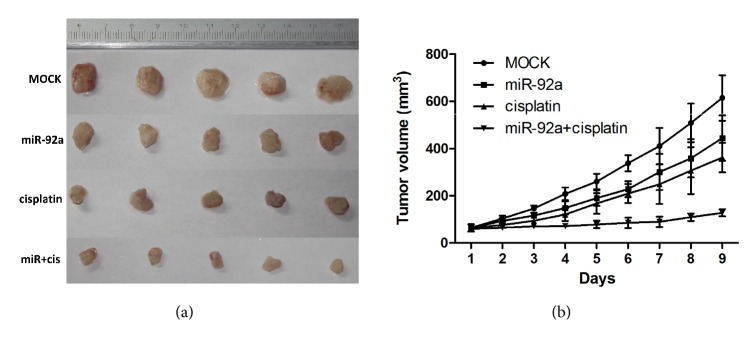
MiR-92a inhibited osteosarcoma tumourigenesis and enhances cisplatin sensitivity in vivo. (a) Representative subcutaneous xenograft from the mice in different group. (b) The growth curve of subcutaneous xenografts of OG63 cells. Data represent mean ± SD. *∗*P < 0.05; *∗∗*P < 0.01.

A mouse xenograft model was established to evaluate the effects of miR-92a on the growth of OS cells and their sensitivity to cisplatin. We observed that miR-92a significantly inhibited OS growth* in vivo*. Moreover, miR-92a overexpression reduced the growth of cisplatin-treated OS tumours compared to that of cells transfected with a miR control (Figures 6(a) and 6(b)).

## 4. Discussion

MiRs have been implicated in the regulation of various physiological and pathological processes, including human cancer [21]. MiR-92a belongs to the miR-17-92 family, which is located on chromosome 13q and encodes six miRs that are processed from a common precursor transcript. MiR-92 has been widely studied and applied in the clinic. For instance, the levels of serum miR-92 may have diagnostic value for differentiating between recurrent and nonrecurrent colon cancer in patients undergoing radical surgery and adjuvant chemotherapy [22]. Higher concentrations of miR-92 were also observed in serum from patients with ovarian epithelial carcinoma compared to healthy controls, and miR-92 levels increased with lymph node involvement and clinical stage of ovarian epithelial carcinoma (EOC) [23].

Owing to its potential use as a biomarker for diagnosis and treatment, it is important to study the mechanism of miR-92a in cancer. In the present study, we explored the function of miR-92a in OS, which is an understudied area. Notably, we found that miR-92a was downregulated in OS tissues and cell lines (MG63 and HOS) compared with the normal tissues and cells. Thus, we next carried out gain- and loss-of-function studies and demonstrated that miR-92a could significantly suppress the proliferation and migration of MG63 and HOS cells and induce G0/G1 arrest. However, their functions are not fully understood.

It is known that miRs regulate gene expression by binding to the 3'-untranslated regions (3'-UTR) of their target mRNAs. We further employed the bioinformatics tool-TargetScan and found that miR-92a directly targeted Notch1 and negatively regulated its protein expression levels in OS cells. Consistently, Notch1 level in OS tissues and cells was significantly elevated compared to those of normal tissues and hFOB cells.

The Notch signalling pathway includes a variety of Notch receptors, such as Notch1, Notch2, Notch3, and Notch4. Recent studies have demonstrated the involvement of Notch ligands, such as Jag1, Jag2, Dll1, Dll3, and Dll4, in the regulation of OS tumourigenesis, progression, and metastasis [24, 25]. The binding of a Notch receptor to its ligand leads to the cleavage of the Notch extracellular domain (NECD) and its transendocytosis into the ligand-expressing cell. The Notch ICD is then released and translocated into the nucleus, where it interacts with transcription factors such as CSL/RBPJk and mastermind-like (MAML) protein. These interactions replace the corepressor complex, activate CSL, and recruit coactivators such as MAML and p300 to initiate the transcription of downstream target genes [26].

Cisplatin is an effective and widely used agent for human solid tumours due to its effectiveness, simple administration and mild side effects. However, cisplatin resistance often occurs in clinical practice, and strategies to promote cancer cell cisplatin sensitivity are urgently needed [27]. Recent studies have shown that miRs contribute to OS chemoresistance [28, 29]. Accumulating evidence also indicates that the Notch signal pathway is involved in chemotherapy resistance in many types of malignant tumours, such as head and neck squamous cell tumours, colorectal tumours, and ovarian cancer [30]. As in OS, it has been reported that the Notch1 signal pathway regulates the sensitivity of OS to cisplatin by modulating the activity of caspases. In our study, we demonstrated that miR-92a enhanced the sensitivity of OS cells to cisplatin. To confirm whether miR-92a exerts this effect through the regulation of Notch1, we performed a rescue experiment in which Notch1 restoration reversed miR-92a-induced cisplatin sensitivity in OS cells.

Our findings may represent novel and reliable target genes for the diagnosis and treatment of osteosarcoma. However, further studies, such as those that investigate interactions between Notch signalling and other pathways, as well as the regulation of downstream genes, are still required.
